# US Primary Care Physician Payments for Productivity and Quality: Trends from Longitudinal National Practice Surveys

**DOI:** 10.1007/s11606-026-10311-y

**Published:** 2026-03-17

**Authors:** Matthew Mackwood, Minah Park, Rachel O. Schmidt, Hector P. Rodriguez, Ellesse-Roselee L. Akré, Alena Berube, Karen E. Schifferdecker

**Affiliations:** 1 Department of Community & Family Medicine, Geisel School of Medicine at Dartmouth, Hanover, NH USA; 2 The Dartmouth Institute, Geisel School of Medicine at Dartmouth, Hanover, NH USA; 3 School of Public Health, University of California, Berkeley, CA USA; 4 Department of Health Policy and Management, Johns Hopkins University Bloomberg School of Public Health, Baltimore, MD USA

## INTRODUCTION

There is an increasing emphasis on payment reform in primary care, driven by efforts to improve quality, control costs, and enhance patient outcomes.^1^ Simultaneously, consolidation in practice ownership and the growth of value-based payment (VBP) models are reshaping the landscape of healthcare delivery. It is unclear how much reforms to shift away from fee-for-service payments and increasing pay for quality are reflected in the compensation primary care physicians receive. To better understand these trends, we analyzed longitudinal data from the two most recent waves of the National Survey of Health Organizations and Systems in 2017–2018 (response rate 43%) and 2022–2023 (response rate 37%).

## METHODS

We conducted a retrospective cohort study of two US national surveys of primary care practice leaders about their practices’ capabilities and characteristics. Among 1959 original survey respondents sampled, 722 (37%) responded to the second survey; of these, 432 practices (60%) answered compensation questions in both and were included in the analysis. Examined characteristics of practices with missing compensation data including ownership, practice size, and VBP model participation were not statistically different from practices with complete compensation data. Our primary outcome was the prevalence of three predominant approaches to compensation: salary, productivity, or both, in each time period. Secondary outcomes were the frequency and amount of compensation for quality (including patient satisfaction). We stratified outcomes by practice ownership structure (independent, physician group, hospital/health system, or Federally Qualified Health Center (FQHC), which disproportionately serves low-income and racially minoritized communities), with the aim of examining changes over time and differences between ownership types. Results were weighted for survey sampling and non-response. Detailed survey methods are described elsewhere.^2^ Stata 17.0 was used for analysis. The Dartmouth College Committee for the Protection of Human Subjects deemed this study exempt.

## RESULTS

Respondent practices are described in Table 1. Overall, compensation primarily through productivity declined by 6% between 2017–2018 and 2022–2023, while salary correspondingly increased; mixed productivity/salary compensation was steady. While over half of practices paid for quality, it represented small proportions of total compensation. FQHCs were far more likely to compensate via salary, and practices paying primarily salary were less likely to pay for quality. There was wide variation in base and quality compensation across all ownership types (Table 2).

**Table 1:** Practice Characteristics, by Ownership Type and Year

Characteristic	Practices, weighted % (unweighted count)			*p*-value for difference between years*
	**Total** **(*n* = 432)**	**Survey year**		
		**2017–2018**	**2022–2023**	
Ownership				< 0.001
Independent		37 (144)	31 (123)	
Group	N/A	13 (63)	10 (47)	
Hospital/health system	N/A	40 (192)	49 (227)	
Federally Qualified Health Center	N/A	10 (33)	10 (35)	
Current ACO^†^ participation				0.79
Yes	N/A	78 (332)	77 (345)	
Physician count				0.68
0–4	N/A	48 (169)	49 (168)	
5–9	N/A	31 (148)	30 (140)	
10–19	N/A	11 (60)	12 (61)	
≥ 20	N/A	9 (55)	9 (58)	
No. of advanced practice professionals in practice^‡†^				0.047
Zero	N/A	20 (83)	18 (80)	
1 or 2	N/A	35 (169)	31 (145)	
3 or 4	N/A	23 (92)	23 (99)	
5 to 10	N/A	13 (53)	19 (61)	
≥ 10	N/A	8 (35)	10 (42)	
Rurality^§^				
Rural	8 (36)	N/A	N/A	N/A
US census region				
New England	7 (39)	N/A	N/A	N/A
Middle Atlantic	11 (43)	N/A	N/A	N/A
East North Central	14 (68)	N/A	N/A	N/A
West North Central	13 (57)	N/A	N/A	N/A
South Atlantic	19 (76)	N/A	N/A	N/A
East South Central	3 (15)	N/A	N/A	N/A
West South Central	8 (29)	N/A	N/A	N/A
Mountain	12 (53)	N/A	N/A	N/A
Pacific	14 (52)	N/A	N/A	N/A

^*^Chi-squared test

^†^ACO, Accountable Care Organization, a type of value-based payment model

^‡^Includes physician assistants and nurse practitioners with specialties of family practice, general practice, internal medicine, or geriatric medicine

^§^Determined by rural-urban commuting area classification for the practice’s business address

**Table 2:** Comparison of Base and Quality Payment Structures by Primary Care Practice Ownership Type

**Payment structure**	**Total (N = 432)**		**Independent**		**Physician group**		**Hospital/health system**		**FQHC**^†^	
	**2017–2018**	**2022–2023**	**2017–2018** **(n = 144)**	**2022–2023** **(n = 123)**	**2017–2018** **(n = 63)**	**2022–2023** **(n = 47)**	**2017–2018** **(n = 192)**	**2022–2023** **(n = 227)**	**2017–2018** **(n = 33)**	**2022–2023** **(n = 35)**
Frequency of base payment type, weighted % of practices (95% CI)*										
Primarily productivity	37 (30–43)	31 (25–37)	46 (34–59)	32 (20–44)	42 (26–59)	44 (26–62)	36 (27–44)	36 (27–44)	10 (4–24)	1 (1–2)
Primarily salary	22 (15–29)	28 (21–35)	18 (9–28)	31 (18–43)	8 (0–15)	18 (1–37)	16 (9–23)	18 (11–24)	70 (47–92)	71 (50–93)
Productivity/salary mix	41 (34–47)	41 (34–48)	35 (23–47)	38 (25–51)	50 (33–67)	38 (20–57)	48 (39–57)	47 (38–55)	21 (3–38)	28 (7–50)
Frequency of bonus pay for quality^‡^, weighted % of practices (95% CI)*										
Total	53 (46–60)	59 (52–66)	44 (31–56)	47 (34–60)	71 (58–84)	53 (34–72)	62 (53–71)	75 (68–82)	25 (4–46)	27 (6–48)
Primarily productivity	63 (53–72)	69 (59–79)	54 (36–71)	57 (35–79)	60 (40–81)	64 (42–87)	71 (59–84)	76 (65–88)	87 (63–100)	0
Primarily salary	21 (9–33)	29 (16–41)	14 (2–26)	30 (6–53)	66 (32–100)	0	31 (10–52)	47 (29–65)	13 (7–33)	14 (5–32)
Productivity/salary mix	60 (50–70)	73 (64–82)	44 (22–66)	53 (32–74)	81 (66–96)	66 (38–93)	66 (53–79)	84 (76–93)	35 (2–68)	62 (25–99)
Proportion of total pay for quality^‡^, weighted % of total compensation (95% CI)*										
Total	6.1 (4.8–7.4)	7.9 (6.6–9.3)	4.1 (2.4–5.9)	6.2 (4.1–8.3)	11.6 (6.2–16.8)	10.6 (2.6–18.4)	6.8 (5.4–8.2)	10.0 (8.4–11.5)	3.2 (0–7.4)	1.8 (0.5–3.2)
Primarily productivity	7.5 (5.5–9.5)	10.1 (7.8–12.3)	5.7 (2.4–9.0)	7.5 (3.9–11.1)	6.2 (3.4–9.0)	8.0 (4.4–11.6)	8.2 (5.9–10.4)	11.8 (8.6–15.0)	27.9 (20.7–35.1)	—
Primarily salary	1.6 (0.8–2.5)	2.6 (1.3–3.8)	1.7 (0.3–3.3)	3.1 (0.6–5.5)	8.1 (3.7–12.4)	—	2.2 (0.7–3.8)	4.6 (2.3–7.0)	0.3 (0–0.8)	0.4 (0–1.0)
Productivity/salary mix	7.0 (4.7–9.3)	10.0 (7.8–12.1)	3.0 (1.4–4.5)	7.6 (3.7–11.5)	16.5 (7.8–25.1)	18.5 (2.4–34.6)	7.0 (4.8–9.2)	10.6 (8.8–12.4)	1.0 (0–2.2)	5.4 (2–8.8)

^*^Results are weighted for survey non-response, with robust standard errors accounting for the sample structure and survey design used to calculate 95% confidence intervals

^†^FQHC = Federally Qualified Health Center

^‡^Includes bonus payments for quality or patient satisfaction

## DISCUSSION

In this retrospective cohort analysis of US primary care practices, we found that over 70% of primary care practices’ compensation still included productivity components despite a modest decline over 5 years, while pay for quality persistently represented less than 10% of pay. Our results highlight stagnation in the shift to emphasize value in primary care physician pay, despite value-based payment reforms.

The finding of a relatively higher prevalence of productivity agrees with one recent study^3^ but differs from the American Medical Association, which aggregated physician responses,^4^ and the Medical Group Management Association, which surveyed only medical groups and health systems.^5^ While our estimates for compensation for quality are somewhat higher, they agree with others that quality comprises a small share of overall pay.

Limitations include an inability to discern dollar amounts; that questions on pay for quality/satisfaction did not ask about shared savings or other compensation mechanisms; potential non-response bias (though we use weights to account for this); a lack of patient demographics in the survey data, limiting equity-oriented analyses; and the possibility for unmeasured confounding, especially for comparisons across ownership types. However, our longitudinal cohort is more robust to such confounding than a serial cross-section and provides evidence of modest changes over time in compensation for primary care physicians across a range of practice types.

Future work is needed to better understand how differences in physician compensation relate to differential care processes and outcomes. Studies of VBP models to date have found heterogeneous impact and underscore the need to distinguish the impact of payments retained by organizations from those reaching individual physicians.^6,7^ Relevant policy efforts, such as state-led initiatives to increase primary care spending targets and investment in payment reforms similar to the recently canceled Making Care Primary innovation model, which sought to advance primary care-focused, prospective, population-based payments, in contrast to the prevailing model of retrospective pay for quality, should provide important opportunities to further study how payment approaches interact with and might best incentivize and support high-quality primary care.

## Data Availability

The data supporting the findings of this study are available from the corresponding author upon reasonable request. The data are not derived from human participants. The full NSHOS I and II survey instruments are publicly available at https://sites.dartmouth.edu/coe/nshos/. Access to the de-identified study data will be granted to qualified researchers upon approval of a research proposal outlining the intended use of the data. Proposals should be submitted to the corresponding author, Karen E. Schiff erdecker (karen.e.schifferdecker@dartmouth.edu). Data will be made available concurrently with the publication of this manuscript.

## Declarations

Generative artificial intelligence such as ChatGPT was NOT used in any stage of ideation, drafting, or revision of this manuscript.
